# Supplementary material for: Chromothripsis during telomere crisis is independent of NHEJ, and consistent with a replicative origin
Source: Genome Res. 2019 May;29(5):737–49. doi: 10.1101/gr.240705.118 (PMC6499312; doi:10.1101/gr.240705.118)
Supplement: Supplemental Material [file supp_gr.240705.118_Supplemental_file_1.zip › contigs/annotated_contigs/DB98/contig.2.DB98_length_432_mean_cov_5.20833333333.docx]

**DB98_length_432_mean_cov_5.20833333333**

```
TAGCAGGGTGTACCAAGCACTCCTGTAGGGCAGGAGCCCCAGGGACATATGTGGAAGCCCATATATCTTGGGGAATATGTGGAAGCGCA
 >chr21:40318701-40318859 + E=1e-80
TATACAAGAAATGATCCCTCTCCTTCCTGGGTGTCTGCACCATGGGACATGCTCTCGGAGGCAGCTTGC|TATATT|TCCGCCCGCATG
                                                                            >chr21:4049
ATCCAACCACCTCCCACCAAGCCCCGCCTCCGACAGTGGGGATTGCAATTCAACGCGAGATTTACAGGAGACGACATCCAAACTATTCT
0329-40490597 + E=7e-144
GCTGGGGGTGGTGTGCATAGCAGGGAAGGAAGGTAGCGTGGATGCAGATGTCCCAGCAGGGTCTCGGATGGGGTTGGAAAGGCCTGTTC

TCTCCCCTAGGAGTAGCCAGGACAGACCAGGCCTGGATGGGACCTGGAAGACACCTCCTGGTTCCCATTACATTTGCA
```
